# Microanalyser Prototype for On-Line Monitoring of Copper(II) Ion in Mining Industrial Processes

**DOI:** 10.3390/s19153382

**Published:** 2019-08-01

**Authors:** Karla Victoria Guevara Amatón, Pedro Couceiro, Hesner Coto Fuentes, Antonio Calvo-Lopez, Natàlia Sández, Héctor Aurelio Moreno Casillas, Francisco Valdés Perezgasga, Julián Alonso-Chamarro

**Affiliations:** 1 Instituto Tecnológico de la Laguna, México, Cuauhtémoc y Revolución s/n, Torreón Coahuila 27000, Mexico; 2 Group of Sensors and Biosensors, Department of Chemistry, Autonomous University of Barcelona, Edifici Cn, 08193 Barcelona, Spain

**Keywords:** copper(II) ion, microanalyzer, multicommutation approach, Nitroso-R-salt, Cyclic Olefin Copolymer

## Abstract

A microanalyzer prototype for copper(II) ion monitoring in mining industrial processes is presented. The microanalyzer is designed as an assembly of different modules, each module being responsible for a unit operation. In order to optimize the industrial processes, the microanalyzer can automate all sample management, signal processing, and mathematical calculations and wirelessly transfer data to a control room. The determination of copper(II) ion is done using a colorimetric reaction and the microanalyser performs autocalibration by in situ dilution of a stock solution, matching the higher analyte concentration of the working range defined for the sample to be determined, using a multicommutation approach. The performance of the microanalyzer for monitoring copper(II) ion in water effluents of mining facilities was optimized in the working range from 1 to 10 mg/L to match Mexican environmental law regulations, which allow a maximum concentration of 4 mg/L of copper(II) ion in these circumstances.

## 1. Introduction

Monitoring of copper(II) ion in water bodies at different stages of copper mining (extraction, preconcentration, electrolysis, wastewater treatment) is of great importance in order to optimize the whole process from an economic and environmental point of view [1]. In this sense, versatile, selective, simple, autonomous, automatic, low cost, on-line, and miniaturized analytical instrumentation with reduced reagent consumption and waste generation is needed. This enables collection of temporal and spatial information about this parameter, and thus, application of the required corrective measures.

Recent trends in analytical chemistry have focused on the integration of the different stages of the analytical procedure (such as sampling, sample transportation and pretreatment, reaction, detection, and signal acquisition and processing) in single systems, which are denoted Total Analysis Systems (TAS) [2,3,4]. In addition, the possibility of miniaturization of some or all of the unit operations has originated the concept of µTAS or lab on a chip (LOC) [5,6,7]. These miniaturized analytical systems present a number of advantages compared to classical analytical instrumentation, which make them ideal for real-time and in situ monitoring of copper(II) ion [8].

Some approaches to determining copper(II) ion in water can be found in the literature [9,10,11,12,13,14,15,16,17,18]. Traditionally, the mining industry has relied on standard atomic absorption spectroscopy (AAS), inductively coupled plasma–optical emission spectrometry (ICP-OES) or inductively coupled plasma mass spectrometry (ICP-MS) for the determination of copper and other metals. Although extremely sensitive and selective, these analytical methods require expensive and complex instruments, trained personal (operators), and controlled laboratory conditions, which make them unsuitable for analysis of aqueous processes. Low cost spectrophotometric methods, using simple instrumentation, with high automation and versatility, based on the use of copper(II) ion selective colorimetric reagents, have been reported. Among them, those that use the chromogenic reagent Nitroso-R salt (NRS) are preferred. In this case, cobalt, iron, and nickel also form colorimetric complexes with NRS [11,12], but an adequate selectivity can be achieved by simply adjusting pH values and selecting the appropriate absorption band λmax, which is characteristic for each metal complex. However, most of the spectrophotometric analyzers described so far do not meet the stringent requirements for on-site operating conditions in the mining industry. Moreover, robust instrumentation, capable of supporting extreme environmental conditions with minimum maintenance, as well as the ability to supply information to the control room in real time, via radio or Wi-Fi, is mandatory for in situ monitoring, approaching the concept of industry 4.0 [19].

In this work, we present a new analyzer prototype for on-line, real-time monitoring of different heavy metals in mining industrial processes. The metal to be analyzed can be selected by simply choosing the appropriate colorimetric reagent used. 

The microanalyzer was designed and fabricated using a versatile modular approach. It allows an easy module replacement in the case of malfunction. Furthermore, it opens the possibility of modulating working conditions in order to meet heavy metal monitoring requirements (sample matrix composition and analyte working range). Robust and miniaturized optical components such as an LED and a photodetector were employed. Advances in optoelectronics in the last decades have allowed new, miniaturized and low cost light sources and detectors to be obtained, such as LEDs and photodiodes, respectively. Thus, the classical complex and expensive instrumentation for optical measurements can be replaced by robust, portable, and low cost instrumentation [20,21,22,23,24]. 

A key feature of the microanalyzer is its multicommutation microflow module, which allows the complete automation of sample/standard solutions management. Automatic sampling and calibration processes can be performed using solenoid microvalves [22,25,26,27,28,29]. The microfluidic platform module, made of cyclic olefin copolymer (COC), integrates micromixing structures and the detection chamber. COC lamination technology offers important advantages such as fast, easy, and cheap fabrication of hermetically sealed, three dimensional microfluidic patterns using economical facilities, high chemical and mechanical resistance, and good transparency in the UV–vis range [24,30,31,32]. The dedicated electronic control module developed for this prototype, based on a programmable system on chip (PSoC), controls all the actuators, the analytical process data acquisition, and wireless communication to the control room, where corrective measures can be applied, if necessary. 

The performance of the microanalyzer is optimized herein for monitoring copper(II) ion in the most demanding operational conditions (mine effluents) at a mining facility located in Mexico. The maximum concentration of copper(II) ion in mine effluents allowed by environmental law regulations in Mexico is 4 mg/L [33]. For this reason, the microanalyzer operational working range was optimized between 1 and 10 mg/L. Wider operational ranges can be easily defined by means of the multicommutation approach to match the composition of samples in other steps of the hydrometallurgical process, if necessary. 

## 2. Materials and Methods

All reagents employed in this work were of analytical grade. Copper standard for ICP, sodium phosphate dibasic (Na_2_HPO_4_), sodium phosphate monobasic (NaH_2_PO_4_), and nitric acid (HNO_3_) were purchased from Sigma-Aldrich. 3-hydroxy-4-nitroso-2,7-naphthalenedisulfonic acid disodium salt (NRS) was purchased from Fluka. Working solutions of stock standard solution of copper(II) ion (10 mg/L), phosphate buffer (pH adjusted to 6.6), NRS reagent, and HNO_3_ (0.1 M) were prepared in MilliQ water and degassed prior to use.

For absorbance measurements, a double-beam scanning spectrophotometer (Shimadzu UV-310PC UV–Vis–NIR, Tokyo, Japan) is used. 

The cyclic olefin copolymer (COC) microfluidic platform module was constructed using 1 mm and 400 μm TOPAS 5013 layers and 25 μm TOPAS 8007 layers from TOPAS Advanced Polymers (Florence, KY, USA).

The miniaturized optical detection module was composed of a compact optical reader [34], fabricated in polymethylmethacrylate (PMMA) (Ferplast, Spain) with integrated housing for a 505 nm LED (Roithner Lasertechnik B5B-433-B505, Farnell, Spain), and a Hamamatsu S1337-66BR photodiode (Farnell, Spain).

The multicommutation microflow module was assembled using three-way solenoid NResearch 161T031 microvalves (NResearch, Switzerland), a Gilson Minipuls 3 peristaltic pump (Gilson, Middleton, WI, USA) with tygon tubing (1.2 mm, Ismatec, Switzerland), and polytetrafluoroethylene (PTFE) (i.d. 0.8 mm, Tecnyfluor, Spain).

The electronic control module was built on a printed circuit board (PCB) designed in-house and built elsewhere (Shenzhen JLC Electronics Ltd., Shenzhen, China). The heart of the electronic control module was a PSoC 5 CY8C5868AXI-LP035 Chip (Cypress Semiconductor, San Jose, CA, USA).

The microanalyzer modules were assembled in an industrial wireless enclosure (Banner BWA-EF14128, Minneapolis, MN, USA).

## 3. Results and Discussion

### 3.1. Microanalyzer Design and Fabrication

**Figure 1:**
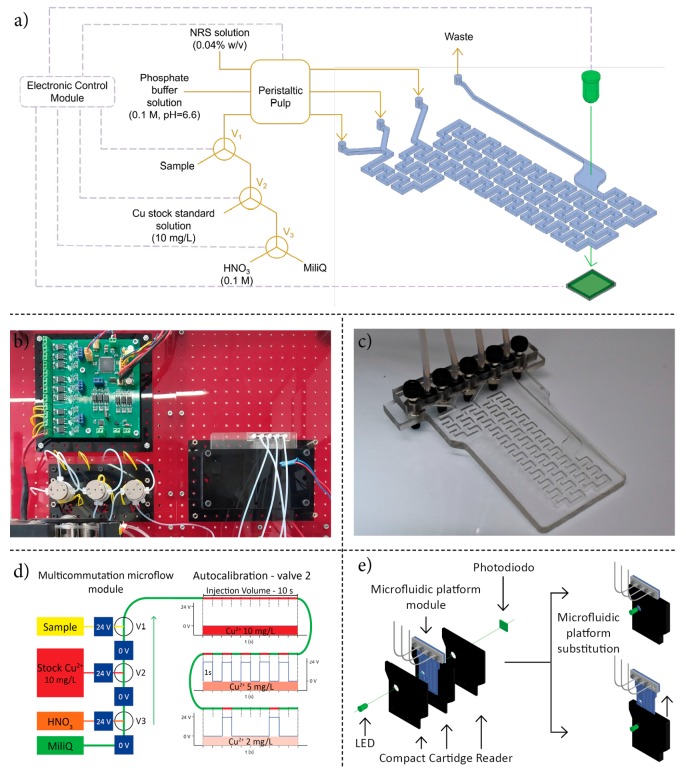
(**a**) Schematic representation of the fluidic components and the microanalyzer (**b**) Photograph of the proposed prototype (**c**) Photograph of the microfluidic platform module (**d**) Schematic representation of the multicommutation microflow module with an autocalibration example (**e**) Schematic representation of the miniaturized optical detection module.

The microanalyzer prototype, shown in Figure 1, was designed as an assembly of four independent modules: a multicommutation microflow module, a miniaturized optical detection module, and a microfluidic platform module, all controlled by an electronic control module and all assembled in an industrial enclosure.

The multicommutation microflow module (Figure 1d) composed of three three-way solenoid microvalves and a peristaltic pump, allows the complete automation of sample/standard solutions management. Using a multicommutation approach, the module can perform an autocalibration by the in situ dilution of one stock solution, containing the highest concentration of the analyte to be determined, using Valve 2, as well as performing autosampling using Valve 1. Valve 3 can be used to perform cleaning steps before the autocalibration process. The use of only one standard solution of high analyte concentration during the calibration process increases the microanalyzer simplicity and its robustness.

The miniaturized optical detection [34] module, seen in Figure 1e, was composed of a compact optical reader with integrated housing for a 505 nm LED, used as the light source, and a photodiode, used as a detector. The compact optical reader, also seen in Figure 1e, incorporated an insertion port for the microfluidic platform module, using a lock and key design. It allowed a perfect alignment between the light source, the microfluidic platform optical detection flow cell, and the photodetector. The miniaturized optical detection module working principle is based on the single-beam measurement concept, since an optical reference was registered before each measurement.

The microfluidic platform module, fabricated using a technological approach previously developed by our research group, has been described in detail elsewhere [35]. The microfluidic platform module was designed with three (3) inlet channels, a serpentine micromixer, an optical detection flow cell, and an outlet channel. The first two (2) inlet channels merge in a Y-shaped confluence point and the fluidic path follows as a serpentine micromixer. A second confluence point connects the third inlet channel with the serpentine micromixer until it reaches the optical detection cell. An outlet channel exits into the waste reservoir. The inlet channel and the serpentine micromixer are of 1.0 mm depth and 0.8 mm width, while the optical detection flow cell is of 1 mm depth (i.e., optical path length) and 4.5 mm diameter. The outlet channel is narrower, 1.0 mm depth and 0.4 mm width, in order to facilitate air bubbles to rise from the optical detection flow cell.

The heart of the electronic control module is a programmable system on chip (PSoC 5, Cypress Semiconductor, San Jose, CA, USA). This is a highly integrated circuit that houses analog and digital programmable blocks including a microcontroller, A/D and D/A modules, amplifiers, filters etc., which can be configured and reconfigured by software to deliver the desired functions. Moreover, PSoC reduces the complexity of the printed circuit board. The photodetector signal is conditioned using a transconductance amplifier with programmable gain followed by a low-pass filter and an additional amplifier. A second analog channel manages the temperature signal of the instrument casing. Both are digitally converted using a delta–sigma A/D 12 bit converter. The transconductance amplifier, all other programmable amplifiers, the filter, and the converter are part of the PSoC as well. The light-emitting diode (LED) is driven by a programmable current source, while the peristaltic pump that drives all solutions is controlled by a voltage generated by a digital to analog converter. Both current and voltage sources are part of the analog block of the PSoC. A program executed by the microcontroller configures amplifier gains, current, and voltage values. The digital block of the PSoC has counters that drive the sequences needed to operate the instrument including the opening and closing of the valves and the control of the peristaltic pump flow rate. A universal asynchronous receiver–transmitter (UART) module is also included as communications interface of the PSoC to an external computer. The PSoC also houses a serial peripheral interface (SPI) module to send the results processed by the microcontroller to external 4–20 mA circuits that convert them to industrial standards.

### 3.2. Chemical and Hydrodynamic Optimization of the Microanalyzer Prototype Variables

Copper extraction from its oxide ores is a multi-step hydrometallurgical process. In order to optimize the extraction process, copper concentration must be controlled for each step. High copper(II) ion concentrations might be found during the acidic ore lixiviation, the preconcentration by solvent extraction, and the electrolytic deposition processes. On the other hand, lower copper(II) ion concentrations might be found when water effluents from the mining process, which are discharged to river basins, are monitored. The final objective of this work was to develop a versatile microanalyzer with analytical features, like detection limit and working range, that can be easily adaptable in order to match specific sample characteristics along the different stages of the copper extraction process. In the present work, the microanalyzer was initially optimized for monitoring copper(II) ion in mine effluent, which is the most demanding stage of the copper monitoring in the mining process. 

**Figure 2:**
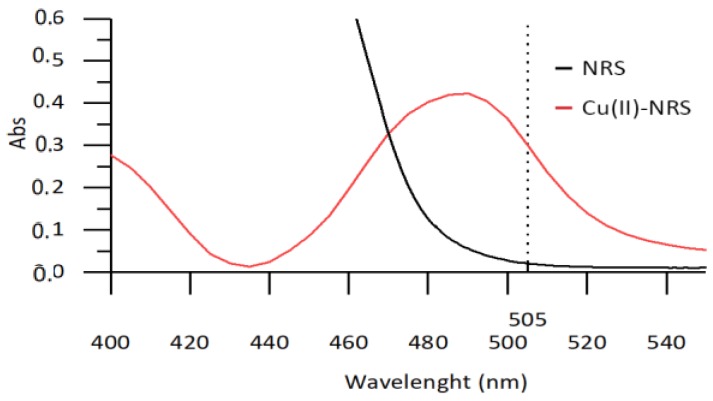
Absorbance spectrum for Nitroso R-Salt (0.04(w/v)) and copper(II)-Nitroso-R complex (10 mg/L).

The chemistry used in the microanalyzer for the determination of copper(II) ion is based on its reaction with NRS reagent to form a colored Cu (II)-NRS complex, the absorbance maximum of which is located at 490 nm and can be measured at the emission wavelength of the LED employed (505 nm), as seen in Figure 2.

The influence of NRS reagent and phosphate buffer concentration in the obtained analytical signal (Abs) of the copper(II)-NRS complex were studied in order to define each optimal concentration. Phosphate buffer pH was adjusted to 6.6 to maximize its buffering capacity. Copper(II) ion and NRS reagent solutions were mixed in a 1:1 ratio, and the absorbance of the resulting copper(II)-NRS complex was measured at 505 nm using a spectrophotometer.

**Figure 3:**
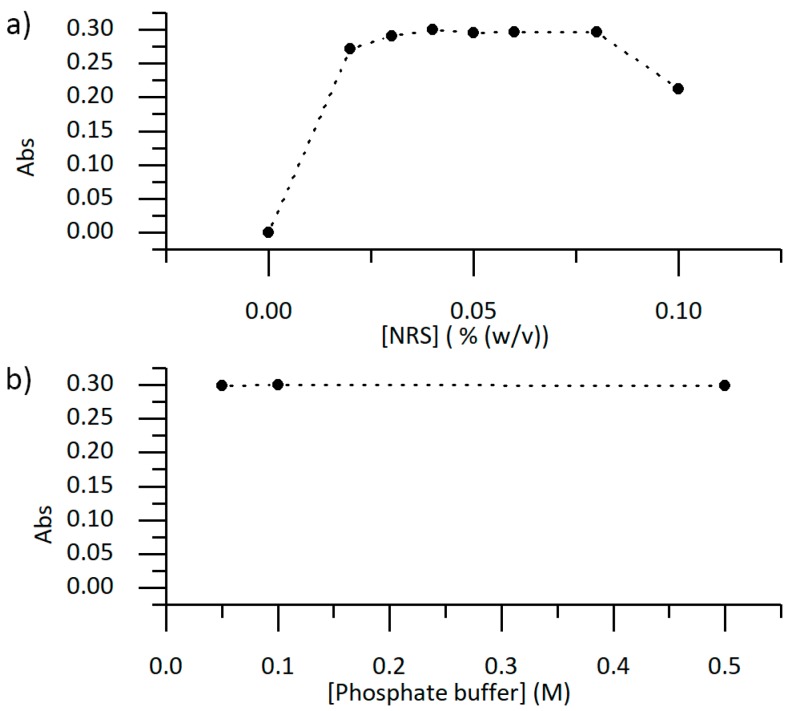
(**a**) Analytical signal (Abs) obtained for a 1:1 ratio mixture of a 10 mg/L copper(II) ion solution in 0.1 M phosphate buffer (pH 6.6), with solutions with increasing concentration of NRS reagent. (**b**) Analytical signal (Abs) for a 1:1 ratio mixture of NRS reagent (0.04% (w/v)) with a 10 mg/L copper(II) ion solution with increased phosphate buffer concentration.

Figure 3a shows that the obtained analytical signal for the copper(II)-NRS complex was maximum when NRS reagent concentration was 0.04% (w/v). For this reason, this concentration was used in all following experiments.

On the other hand, the analytical signal variation of the copper(II)-NRS complex with the concentration of phosphate buffer concentration was negligible (Figure 3b) for the concentration range studied. Therefore, a concentration of 0.1 M of phosphate buffer was used in all following experiments.

**Figure 4:**
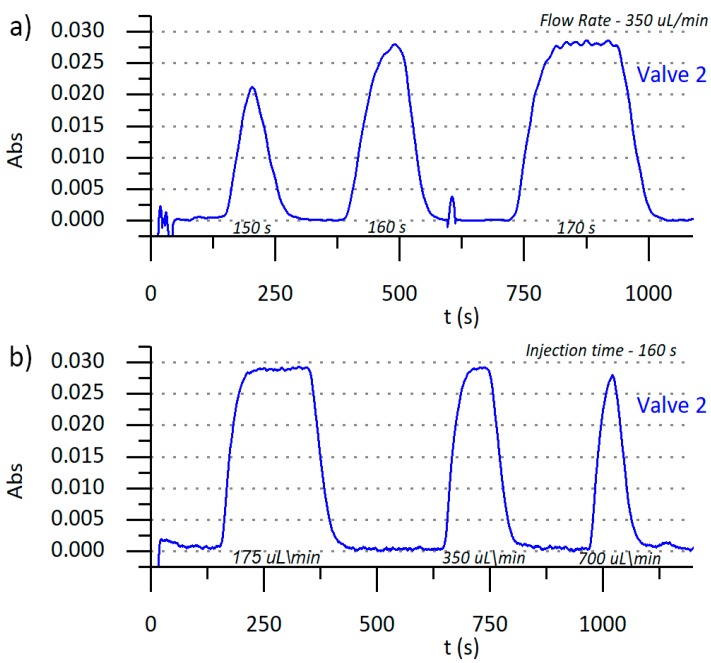
(**a**) Analytical signal (Abs) obtained for increasing injection times at a constant flow rate of 350 uL/min for Valve 2. (**b**) Analytical signal obtained for increasing flow rates at a constant injection time of 160 s for Valve 2.

Using the reagent concentration determined in the previous section, the influence of hydrodynamic parameters of the microanalyzer, like injection volume and flow rate, were studied using a univariate optimization procedure. In order to obtain robust operational characteristics of the microanalyzer, steady state signal conditions should be used whenever possible. Since the analytical signal will not vary over time in these working conditions, fluctuations in the analytical signal related to small variations in the injection volumes caused by mechanical imperfections of the actuators, will be negligible. In a multicommutation flow system, for a constant flow rate, the injection volume is dependent on the valves’ actuation time, and, therefore, the injection time must be studied in order to determine the range at which a steady state analytical signal is obtained. Figure 4 shows that for a 350 µL/min flow rate, this was achieved at injection times larger than 160 s. These conditions were used to ensure the analytical signal stability at steady state conditions in 30 s.

**Figure 5:**
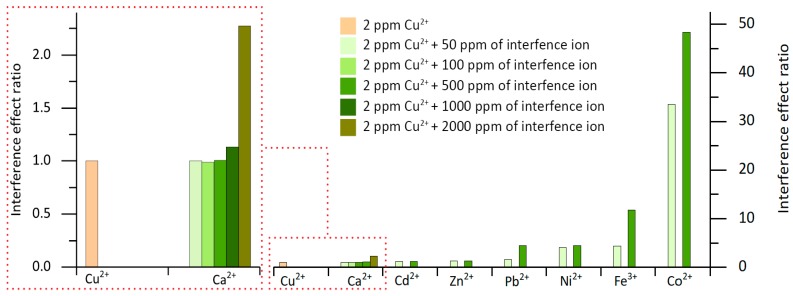
Ion interference effect ratio, expressed as (analyte signal + interference signal)/analyte signal.

The interference effect of ions in the colorimetric determination of copper(II) ion using NRS reagent was also studied, and the results are shown in Figure 5. The interference effect ratio, expressed as (analyte signal + interference signal)/analyte signal, showed no significant interference of the ions studied, except for iron(III) and cobalt(II) ions. However, it is not expected that significant cobalt(II) ion concentrations will be found in copper mine effluents. Since Ca(OH)_2_ is commonly used to neutralize low pH values in mine drain water coming from the acidic lixiviation process, iron(III) is expected to be precipitated during this neutralization process. Therefore, no significant concentration of iron(III) was expected to be present in the sample. The influence of calcium(II) ion was also studied in the 0 to 2000 mg/L range. As seen in Figure 5, no interference effect of this ion in concentrations lower than 1000 mg/L was observed.

Since steady state signal was reached for injection times greater than 160 s (933 µL) and when choosing 1 s for the multicommutation actuation time of the solenoid valves, the microanalyzer was programmed to perform an autocalibration using the parameters depicted in Table 1.

**Figure 6:**
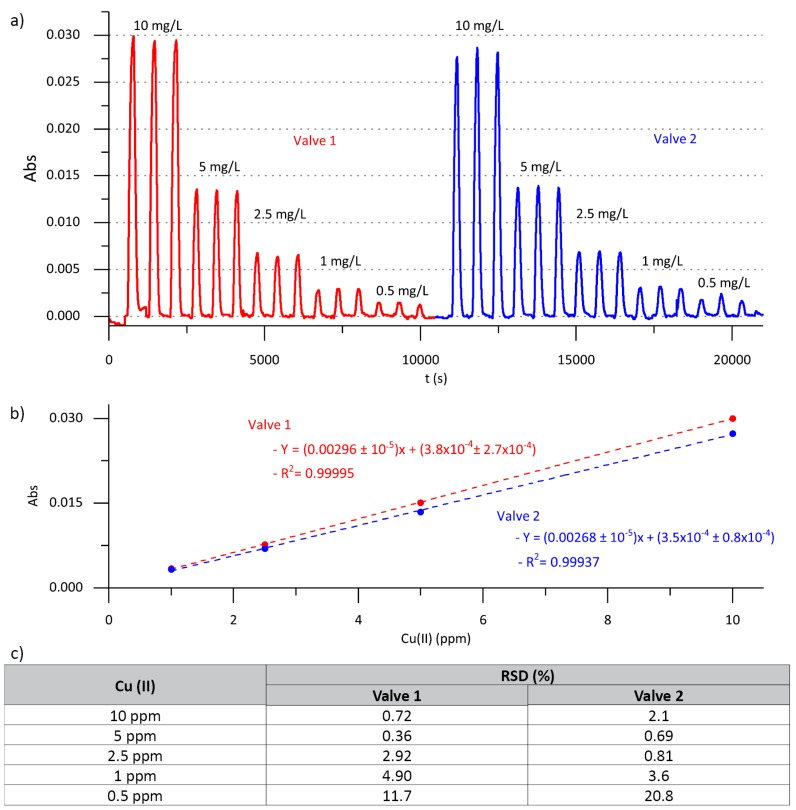
(**a**) Autocalibration performed using Valve 1 and Valve 2. (**b**) Calibration curves obtained for the autocalibration of Valve 1 and Valve 2. (**c**) Relative standard deviation (RSD) values obtained for the triplicate injection of each calibration solution obtained for Valve 1 and Valve 2.

As seen in Figure 6, autocalibration of the microanalyzer was carried out using triplicate injection of standard solutions in the 10 mg/L to 0.5 mg/L copper(II) ion concentration range, obtained by in situ dilution using multicommutation, for both Valve 1 and Valve 2. For the same hydrodynamic conditions, the peak height was slightly smaller for injections using Valve 2 than for injections using Valve 1. This is due to the longer fluidic path, and consequently higher dispersion of the injection plug when the Valve 2 was used. However, no significant differences were obtained for the autocalibration using different valves, as expected. Linear responses were obtained with R^2^ > 0.999 for both valves. The consecutive injections of standard solutions of 10, 5, 2.5, and 1 mg/L of copper(II) ion showed a relative standard deviation (RSD) < 5%, demonstrating the reproducibility of the proposed prototype in this working range. The high RSD values obtained with both valves for the in situ generation of 0.5 mg/L sample were probably the consequence of the slow actuation time used for the multicommutation of the solenoid valves. This problem can be easily solved by reducing the actuation time of the solenoid valves, thus facilitating the in situ dilution of the stock solution. This optimization was, however, not necessary for this specific application. 

Applying the IUPAC definition, the microanalyzer detection limit (LOD) was 0.06 mg/L of copper(II) ion. 

### 3.3. Microanalyzer Programable Sequence

**Figure 7:**
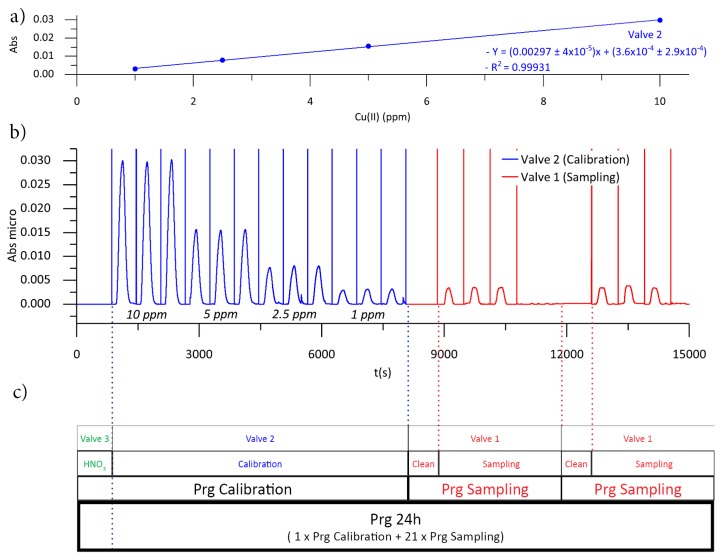
(**a**) Calibration curve obtained using Prg Calibration. (**b**) Analytical signal obtained with Prg 24 h. (**c**) Schematic representation of the Prg 24 h.

A programming sequence called Prg 24 h, consisting of an autocalibration program (Prg Calibration), with a duration of 8100 s (2 h 15 min) and 21 autosampling programs (Prg Sampling), each with a duration of 1 hour, was implemented. As seen in Figure 7c, Prg Calibration starts with a cleaning step of the microanalyzer by injection of HNO_3_ through the actuation of Valve 3, followed by an autocalibration, prepared in situ by multicommutation of Valve 2.

The microanalyzer can be programmed to register, at a specific time, reference values, shown in Figure 7b as the vertical lines between peaks. Reference values have a double function: they allow the miniaturized optical detection module to function as a single-beam photometer, eliminating photodetector voltage drifts due to temperature variations, and they define the data to be processed mathematically by the PSoC. The PSoC is programed to calculate the local maximum between two reference values. It is thus possible to program the microanalyzer in a complex way, defining the injection of cleaning reagents, i.e., HNO_3_, at the beginning of the Prg Calibration, which will not be mathematically processed by the PSoC because it is not framed between two reference values.

Prg Sampling was composed of four real sample injections, using Valve 1. The first injection contained no reference value, and thus it was not mathematically processed by the PSoC, and allows cleaning and refreshing of the solution in the sampling tube, ensuring that representative sampling is obtained. The hydrodynamic parameters of the cleaning sampling tube stage depend on the distance of the microanalyzer to the actual sample collection point, so they must be optimized for any application in the field. The next three sample injections were triplicate injection of real sample and were used to calculate the copper(II) ion concentration.

The mean value of each real sample triplicate analysis, obtained using Prg Calibration, was interpolated into the linear regression in order to calculate the concentration of copper(II) ion. As seen in Figure 7a, linear responses between Abs and the copper(II) ion concentration were obtained, with R^2^ > 0.999, in the 10 mg/L to 1 mg/L copper(II) ion concentration range, with RSD < 5%, demonstrating the reproducibility of the microanalyzer. Different concentration ranges can be easily obtained using the multicommutation approach in order to match the composition of samples in other stages of the copper mining process, if necessary.

## 4. Conclusions

A microanalyzer prototype for copper(II) ion monitoring, using a colorimetric reaction, in mining industrial processes was designed and evaluated. In a laboratory environment, linear responses between analytical signal and copper(II) ion concentration were obtained, with R^2^ > 0.999 in the 10 mg/L to 1 mg/L copper(II) ion concentration range. Moreover, a RSD lower than 5% was obtained, demonstrating the reproducibility of the microanalyzer.

In order to validate the obtained results, the microanalyzer prototype will be installed in the near future in a mining industrial facility for copper(II) ion monitoring in real world experimental conditions.

## Figures and Tables

**Table 1:** Multicommutation algorithm used for in situ preparation of autocalibration dilution, using a single 10 mg/L copper(II) stock solution.

Cu (mg/L)	Dilution (1/n)	Valve Position		
		Stock [Cu] = 10 mg/L	H_2_O (MiliQ)	Cycles
10	1	160 s	0	×1
5	1/2	1 s	1 s	×80
2.5	1/4	1 s	3 s	×40
1	1/10	1 s	9 s	×16
0.5	1/20	1 s	19 s	×8
